# Use of routinely collected data in a UK cohort of publicly funded randomised clinical trials

**DOI:** 10.12688/f1000research.23316.3

**Published:** 2021-03-12

**Authors:** Andrew J. McKay, Ashley P. Jones, Carrol L. Gamble, Andrew J. Farmer, Paula R. Williamson

**Affiliations:** 1 Liverpool Clinical Trials Centre, University of Liverpool, a member of Liverpool Health Partners, Liverpool, UK; 2 MRC North West Hub for Trials Methodology Research, Department of Biostatistics, University of Liverpool, Liverpool, UK; 3 Nuffield Department of Primary Care Health Sciences, University of Oxford, Oxford, UK

**Keywords:** Electronic Health Records, Data linkage, EHR, NIHR HTA, Randomised Clinical Trial, Randomised Controlled Trial, RCT, Registry, Routinely collected data, Routinely collected health data, RCHD

## Abstract

Routinely collected data about health in medical records, registries and hospital activity statistics is now routinely collected in an electronic form. The extent to which such sources of data are now being routinely accessed to deliver efficient clinical trials, is unclear. The aim of this study was to ascertain current practice amongst a United Kingdom (UK) cohort of recently funded and ongoing randomised controlled trials (RCTs) in relation to sources and use of routinely collected outcome data.

Recently funded and ongoing RCTs were identified for inclusion by searching the National Institute for Health Research journals library. Trials that have a protocol available were assessed for inclusion and those that use or plan to use routinely collected health data (RCHD) for at least one outcome were included. RCHD sources and outcome information were extracted.

Of 216 RCTs, 102 (47%) planned to use RCHD. A RCHD source was the sole source of outcome data for at least one outcome in 46 (45%) of those 102 trials. The most frequent sources are Hospital Episode Statistics (HES) and Office for National Statistics (ONS), with the most common outcome data to be extracted being on mortality, hospital admission, and health service resource use.

Our study has found that around half of publicly funded trials in a UK cohort (NIHR HTA funded trials that had a protocol available) plan to collect outcome data from routinely collected data sources.

## Introduction

Routinely collected data about health in medical records, registries and hospital activity statistics is now routinely collected in an electronic form. Progress in achieving connectivity, data linkage and security now offers the possibility of better use of this data for research purposes. For example, recent evidence shows the utility of long-term follow-up of trial patients by linkage to routinely collected health data (RCHD) sources ( Fitzpatrick *et al.*, 2018). Innovative data-enabled study designs can answer pressing knowledge gaps in research evidence. However, the extent to which such sources of data are now being routinely employed in research to deliver efficient clinical trials, potentially at a wide scale, is unclear.

The aim of this study was to ascertain current practice amongst a United Kingdom (UK) cohort of recently funded and ongoing randomised controlled trials (RCTs) in relation to sources and use of routinely collected outcome data. We chose NIHR HTA because they are a major source of funding for investigator-led publicly funded clinical trials within the UK in an NHS setting. We define RCHD to be data collected without specific *a priori* research questions developed prior to using the data for research.

## Methods

### Inclusion criteria

The following inclusion criteria were used:

1. Ongoing RCT of any type including feasibility or pilot work, funded by the National Institute for Health Research (NIHR) Health Technology Assessment (HTA) programme;
2. availability of a protocol; and
3. use of RCHD for at least one study outcome.

### Search methods

A search of the NIHR Journals Library was undertaken to find protocols registered as of 25/10/2019. The search fields and terms used to select were:

1. Search term: ‘Random’
2. Research type: ‘Primary research’
3. Programme: ‘HTA’
4. Status: ‘Research in progress’

If the final published report was shown alongside the protocol this was taken to mean that the RCT was not ongoing but the status had not been updated to ‘Published’, and the study was excluded.

In the absence of a protocol, the study was excluded. For studies with multiple protocol versions, the most recently available version was used.

### Data extraction

One person (AM) extracted the information and categorised each RCHD source, with a second person (PW) checking classifications and explanations. The information extracted was as follows: Lead Investigator surname, year started, ISRCTN, project title, study type, use of RCHD for at least one study outcome, availability of a protocol, any details of data quality assessment of RCHD source prior to use, RCHD source name, reasons for wanting outcome data from RCHD source, specific outcomes and outcome type where clear data to be used will come from named RCHD sources.

## Results

**Figure 1.**
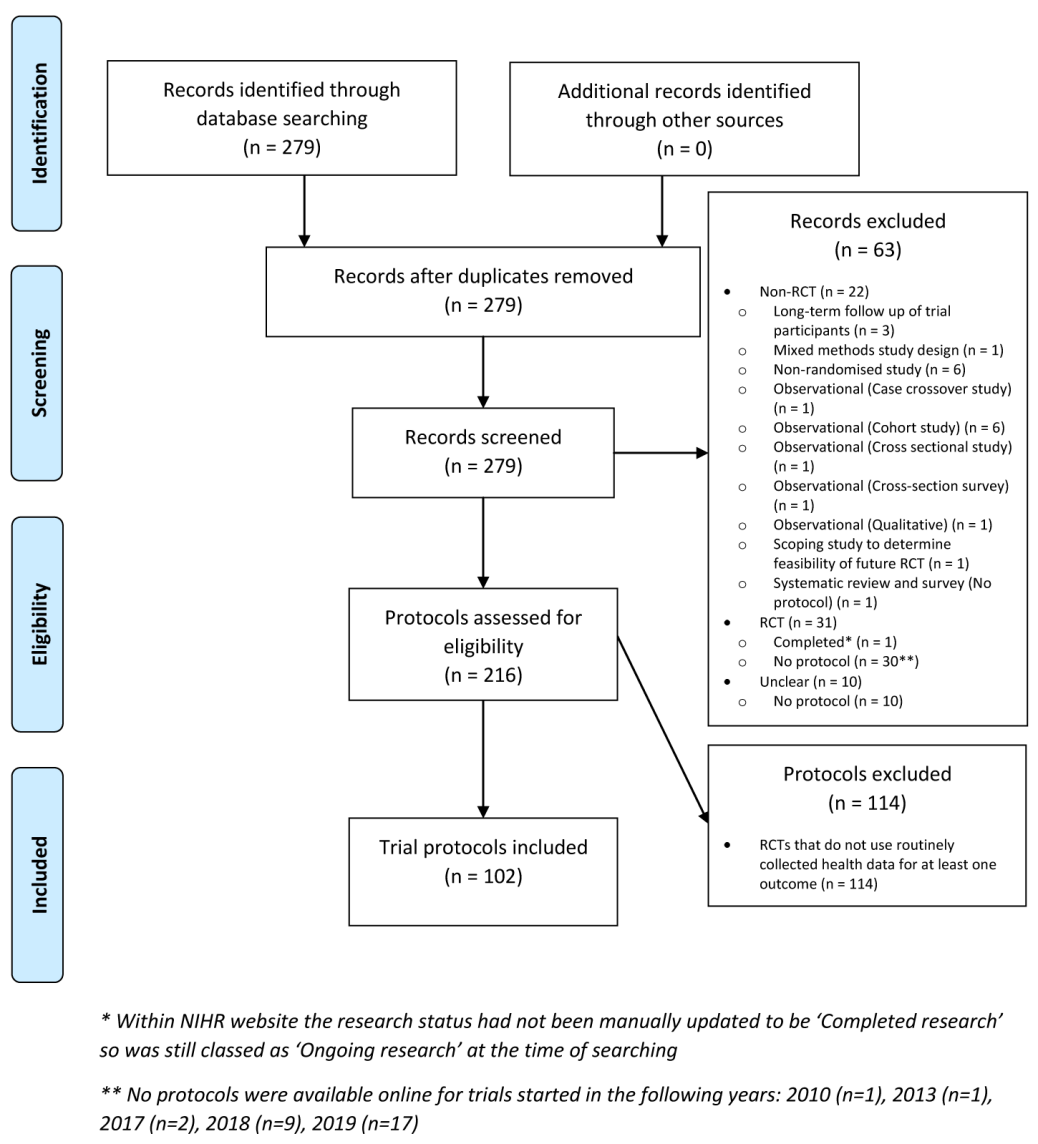
PRISMA flow diagram.

Figure 1 shows the study flow diagram. 279 records were identified through database searching and screened for inclusion. 22 were non-RCTs, 1 was a completed RCT, 30 were RCTs but no protocol was available and 10 were unclear. Of the remaining 216 NIHR HTA trials with a protocol available for further study, 102 (47%) planned to use RCHD for at least one outcome.

Table 1 shows the reasons for collecting trial outcome data from routine sources from the 102 eligible trials. The RCHD source was the sole source of outcome data for at least one outcome in 46 (45%) of those 102 trials (categories 3, 4 and 6 in Table 1). In five of these 46 protocols there was reference to prior feasibility work confirming aspects of the quality of the data to be sufficient for the main trial. Of the 102 trials, 14 (categories 7a-7d in Table 1) planned to assess the feasibility of using the RCHD sources during the trial, although details of the assessment were often lacking. Raw data for Figure 1 and Table 1 and Table 2 are available (see *Underlying data*, McKay *et al.* (2020)).

**Table 1.** Reasons for sourcing outcome data from RCHD sources in 102 studies. Multiple categories can apply to a single study.

	Categories	Total
(1)	(1a) 'Supplementing data collection for withdrawn patients (consent asked for at time of withdrawal)'	7
	(1b) 'Supplementing data collection for lost-to-follow-up patients'	8
	(1c) 'Supplementing data collection for withdrawn patients (consent NOT ASKED FOR at time of withdrawal)'	2
	(1e) 'Continued data collection for withdrawn patients (consent asked for at time of withdrawal)'	1
(2)	(2) 'Supplementing data collection for unobtainable/missing data'	3
(3)	(3a) 'As the sole source of all outcome data'	0
	(3b) 'As the sole source of all outcome data except for data related to protocol adherence and adverse event reporting being collected using CRFs'	0
(4)	(4) 'As the sole source of some outcome data'	43
(5)	(5a) 'As a source of some outcome data, alongside other sources for the same outcome data (e.g. CRF)'	51
	(5b) 'As a source of some outcome data, but collected by CRF if unable to access data'	3
(6)	(6a) 'Registry trial *: As the sole source of outcome data with purpose-built Module to collect remaining outcome data'	1
	(6b) 'Registry trial *: All outcome data collected through multiple RCHD sources except for questionnaire data'	1
	(6c) 'Registry trial *: All outcome data collected through multiple RCHD sources except for some baseline data, questionnaire data and other patient-reported data'	1
(7)	(7a) 'RCHD compared to trial collected data as part of feasibility assessment criteria'	11
	(7b) 'RCHD compared to trial collected data as a main trial secondary outcome'	1
	(7c) 'RCHD compared to trial collected data and then collect long-term follow-up data as part of trial'	1
	(7d) 'RCHD compared to trial collected data and then collect long-term follow-up data after trial has been completed'	1
	(7e) 'Representativeness of randomised patients compared with all eligible patients using RCHD as part of feasibility assessment criteria'	1
(8)	(8a) 'Participants flagged with NHS Digital/other: Check health status of patient prior to contacting in case patient has died'	2
	(8b) 'Participants flagged with NHS Digital/other: Check health status/notification of any deaths, causes'	12
(9)	(9) 'Set up mechanisms for long-term follow-up'	4
(10)	(10) 'Patients asked to provide written consent for continuation in the study once have regained capacity. Those who prefer not to be actively involved in the study follow-up, then asked to provide consent to using their routinely collected NHS data'	1
	**Total**	**155**

* A registry trial is a RCT conducted using clinical observational registries as the main source of outcome data collection

**Table 2.** Categories of RCHD sources of outcome data in 46 studies where this was the sole source for at least one outcome.

Source	Number (%)
(i) Primary care data (all regional equivalents)	8 (17%)
(ii) HES (and/or regional equivalents)	27 (59%)
(iii) ONS (and/or regional equivalents)	27 (59%)
(iv) Data collected specifically for patient group or healthcare intervention (to include patient registries, ICNARC, ambulance, etc)	26 (57%)
(v) Other	5 (11%)

Table 2 shows the RCHD sources of outcome data to be used in these 46 studies. The most frequent RCHD sources are Hospital Episode Statistics (HES) and Office for National Statistics (ONS), with the most common outcome data to be extracted being on mortality, hospital admission, and health service resource use (see *Underlying data*, Data Set 5; McKay *et al.* (2020)). The full list of RCHD sources is given in *Extended data*, Supplementary Table 1 ( McKay *et al.*, 2020).

## Discussion

Our study has found that around half of publicly funded trials in a UK cohort (NIHR HTA funded trials that had protocol available) plan to collect outcome data from RCHD sources. A cohort of 189 RCTs published since 2000, the majority of which were carried out in North America ( McCord *et al.*, 2019), found this figure to be higher at 84%, however, they identified their cohort as those mentioning ‘EHR’ in some way, i.e. a selected cohort, whereas ours is an unselected cohort so they are not comparable due to the selectivity of the samples.

Very few trial teams described any assessments of data quality from RCHDs in the protocol. Work is ongoing that should determine whether such information should be reported in the trial publication ( Kwakkenbos *et al.*, 2018). An extension to the SPIRIT guidelines for trials using RCHD is soon to be initiated, and will determine whether this information should be included in the trial protocol. As a minimum, it is recommended that trialists provide evidence in any funding application about the quality of the data from the RCHD source.

## Data availability

### Underlying data

Figshare: Use of routinely collected data in a UK cohort of publicly funded randomised clinical trials. https://doi.org/10.6084/m9.figshare.12185193 ( McKay *et al.*, 2020).

This project contains the following underlying data:

- Data_Set_1_Details_and_Figure_1_v1.0.csv. (Study identifiers and raw data used for Figure 1.)
- Data_Set_2_Table_1_v1.0.csv. (Raw data used for Table 1.)
- Data_set_3_Supp_Table_1_v1.0.csv. (Raw data used for Supplementary Table 1.)
- Data_set_4_Table_2_v1.0.csv. (Raw data used for Table 2.)
- Data_set_5_Outcomes_using_EHR_data_v1.0.csv. (Raw data showing details of outcomes using data from RCHD sources.)

### Extended data

Figshare: Use of routinely collected data in a UK cohort of publicly funded randomised clinical trials. https://doi.org/10.6084/m9.figshare.12185193 ( McKay *et al.*, 2020).

This project contains the following extended data:

- Supplementary Table 1 - EHR sources of outcome data v1.0.pdf. (Supplementary Table 1.)

Data are available under the terms of the Creative Commons Zero “No rights reserved” data waiver (CC0 1.0 Public domain dedication).
