# Supplementary material for: Use of routinely collected data in a UK cohort of publicly funded randomised clinical trials
Source: F1000Res. 2021 Mar 12;9:323. Originally published 2020 May 4. [Version 3] doi: 10.12688/f1000research.23316.3 (PMC7607478; doi:10.12688/f1000research.23316.3)
Supplement: Peer review history

# Peer review: Reviewer response for version 2

**DOI:** 10.5256/f1000research.26780.r72610

**Published:** 2020-11-02

**Authors:** Merrick Zwarenstein, Alison Howie

**Affiliations:** 1 Western University, London, ON, Canada; 2 Centre for Studies in Family Medicine, Department of Family Medicine, Western University, London, Canada

A sentence could be included to explain your rationale for selecting trials from the Health Technology Assessment Programme at NIHR as against some other source of publicly funded trials. For example your study excludes UKMRC funded trials. The implications of excluding these mechanism-of-action and therapeutic intervention trials could be explained in the discussion.   

 Your flow diagram indicates that you identified articles through database searching (NIHR library) (n=279) and other sources (n=0). If NIHR HTA trials were of interest, what “other sources” were searched, and why? 

 Table 2 includes an “other” category for sources of outcome data. It only includes 5 studies, so  perhaps you could list these “other” sources, in an appendix if necessary. 

 You mention that McCord *et al.* found that 8% of trials published since 2000 used outcome data from RCHD sources.  

- The McCord paper states, “In most (84%) of the trials in our sample, outcomes were measured with the use of EHRs”. It’s not entirely clear where the 8% is from? 

- I think it’s important to note that they searched up to 2017; thus, since your study included trials up to almost the end of 2019, the higher percentage you found may represent a shift over time, especially as the appreciation for pragmatic trials and routinely collected data for these trials has increased in recent years. 

Is the work clearly and accurately presented and does it cite the current literature?

Yes

If applicable, is the statistical analysis and its interpretation appropriate?

Yes

Are all the source data underlying the results available to ensure full reproducibility?

Yes

Is the study design appropriate and is the work technically sound?

Yes

Are the conclusions drawn adequately supported by the results?

Yes

Are sufficient details of methods and analysis provided to allow replication by others?

Yes

Reviewer Expertise:

Randomized trials, esp pragmatic trials; Epidemiology.

We confirm that we have read this submission and believe that we have an appropriate level of expertise to confirm that it is of an acceptable scientific standard.

# Response

**Published:** 2021-02-26

**Authors:** Andrew McKay

**Affiliations:** University of Liverpool, UK

Thanks for your important comments. We have made some changes within the article update to version 3.

**Re Comment 1:**

 We have added a sentence added to introduction:

 “ *We chose NIHR HTA because they are a major source of funding for investigator-led publicly funded clinical trials within the UK in an NHS setting.*”

**Re Comment 2:**

 No other sources were searched. The ‘other sources’ box is part of the ‘PRISMA 2009 Flow Diagram’ template.

**Re Comment 3:**

 These “other” sources are already listed in Supplementary Table 1 as follows:

 (1) - Costa (2016a)

 “ *Linkage to routine NHS datasets*”

 (2) - Morris (2018)

 “ *Central UK NHS bodies for long-term outcomes*”

 (3) - Ramnarayan (2019)

 “ *Data linkage with routine sources (e.g. NHS Digital or equivalent)*”

 (4) - Tickle (2017)

 “ *Routinely collected data held by the Information Services Division (ISD) of NHS Scotland, Business Services Authority (BSA, England) and Business Services Organisation (BSO, Northern Ireland)*”

 (5) - Toff (2013)

 “ *Central NHS databases administered by the Health and Social Care Information Centre and its counterparts in the devolved nations*”

 The text already references this re Table 2 in the Results section: “ *…The full list of RCHD sources is given in* *Extended data* *, Supplementary Table 1 ( McKay *et al.*, 2020).*”

**Re Comment 4a:**

 Thank you for spotting this error – the 8% should read 84%. On reflection, we don’t think the McCord 2019 ‘cohort’ is a similar type of cohort to ours so have amended the text.

 We have removed the text regarding this from the abstract and changed the text within the Discussion to say:

 “ *A cohort of 189 RCTs published since 2000, the majority of which were carried out in North America (McCord et al., 2019), found this figure to be higher at 84%, however, they identified their cohort as those mentioning ‘EHR’ in some way, i.e. a selected cohort, whereas ours is an unselected cohort so they are not comparable due to the selectivity of the samples.*”

**Re Comment 4b:**

 We have checked the data and can confirm that there has been no shift over time re trials that use RCHD for at least one study outcome:

 Trials started <=2017: 79/166=(47.6%)

 Trials started 2018-2019: 23/50=(46.0%).

# Peer review: Reviewer response for version 2

**DOI:** 10.5256/f1000research.26780.r64143

**Published:** 2020-06-02

**Authors:** Sharon Love

**Affiliations:** 1 MRC Clinical Trials Unit, University College London, London, UK

Thank you for making the suggested changes. I have no further comments.

Is the work clearly and accurately presented and does it cite the current literature?

Yes

If applicable, is the statistical analysis and its interpretation appropriate?

Partly

Are all the source data underlying the results available to ensure full reproducibility?

Yes

Is the study design appropriate and is the work technically sound?

Yes

Are the conclusions drawn adequately supported by the results?

Partly

Are sufficient details of methods and analysis provided to allow replication by others?

Yes

Reviewer Expertise:

Trial conduct, particularly monitoring and the use of routinely collected health data.

I confirm that I have read this submission and believe that I have an appropriate level of expertise to confirm that it is of an acceptable scientific standard.

# Peer review: Reviewer response for version 1

**DOI:** 10.5256/f1000research.25738.r63052

**Published:** 2020-05-13

**Authors:** Sharon Love

**Affiliations:** 1 MRC Clinical Trials Unit, University College London, London, UK

This is a review of the protocols of RCTs, currently in progress, funded by NIHR, UK. RCTs were selected from NIHR HTA funding stream list if they claimed to be using routinely collected data for at least one study outcome. The authors found 102 trial protocols matching this criteria and from data extraction that 46 of these were using routinely collected data solely for at least one outcome. The research also found that a handful referenced previous feasibility work confirming the quality of the EHR and also gives a useful table categorising for the 102 trials how they used EHR.

**Major Comments**

 I have only one major comment and it is the reason for both the ‘partly’ options below. The sample was selected to be using routinely collected data for at least one study outcome. Therefore I think the main result should contain this information. I consider that “in a UK cohort” is not enough of a description of the cohort. The fact that the sample was selected based on using routine data for an outcome is crucial in the interpretation.

 The main result is that of 102 protocols using routinely collected data for an outcome, 46 were using routinely collected data as their sole source for at least one outcome. 46/102=45%. Around a half of NIHR HTA funded trials that had an uploaded protocol and used routinely collected health data for at least one study outcome, used solely routinely collected data for at least one trial outcome.

 I think this is an important result.

**Minor comments**

1. Abstract – last part of the last sentence has a word missing “The majority of **which** were carried out in North America”.
2. If you have space in the text, it would be useful to add the information that 30 were omitted due to not having a protocol.
3. The flow chart shows you selected the papers by selecting RCT, those that had a protocol and then those using routinely collected data for at least one outcome. I would be tempted to list the inclusion criteria in the paper in the same order.
4. The second inclusion criteria is “use of routinely collected health data”. Elsewhere you use the term EHR. I would be tempted to be consistent.
5. Table 1: category 10 description appears incomplete.
6. Table 1: could you add a footnote of the definition of a registry trial?

Is the work clearly and accurately presented and does it cite the current literature?

Yes

If applicable, is the statistical analysis and its interpretation appropriate?

Partly

Are all the source data underlying the results available to ensure full reproducibility?

Yes

Is the study design appropriate and is the work technically sound?

Yes

Are the conclusions drawn adequately supported by the results?

Partly

Are sufficient details of methods and analysis provided to allow replication by others?

Yes

Reviewer Expertise:

Trial conduct, particularly monitoring and the use of routinely collected health data.

I confirm that I have read this submission and believe that I have an appropriate level of expertise to confirm that it is of an acceptable scientific standard, however I have significant reservations, as outlined above.

# Response

**Published:** 2020-05-19

**Authors:** Andrew McKay

**Affiliations:** University of Liverpool, UK

Major comments: Thanks for your important comments. We have made these clearer within the article update to version 2.

Major comments part 1: We have now made it clear that the “UK cohort” is “NIHR HTA trials with a protocol” ongoing at the stated data extraction date.

Major comments part 2: We have now made this clearer.

Minor comments: Thank you for your comments. We have addressed them all within the article update to version 2. In relation to one specific comment, we have chosen to use ‘routinely collected health data (RCHD)’ throughout rather than ‘Electronic Health Record (EHR)’ for consistency.
